# Multivariate sea storm hindcasting and design: the isotropic buoy-ungauged generator procedure

**DOI:** 10.1038/s41598-020-77329-y

**Published:** 2020-11-25

**Authors:** Gianfausto Salvadori, Giuseppe Roberto Tomasicchio, Felice D’Alessandro, Letizia Lusito, Antonio Francone

**Affiliations:** 1 Dipartimento di Matematica e Fisica, Università del Salento, 73100 Lecce, Italy; 2 Dipartimento di Ingegneria dell’Innovazione, Università del Salento, 73100 Lecce, Italy; 3 Dipartimento di Scienze e Politiche Ambientali, Università degli Studi di Milano, 20133 Milan, Italy; 4 Dipartimento di Ingegneria Civile, Ambientale, del Territorio, Edile e di Chimica, Politecnico di Bari, 70125 Bari, Italy

**Keywords:** Ocean sciences, Mathematics and computing

## Abstract

The present work provides indications for assessment of wave climate and design of structures at sea at ungauged sites, both critical issues in Ocean sciences. The paper is of methodological nature and of global worldwide applicability. It shows how suitable wave hindcasting relations can be exploited in order to provide sea storm scenarios at an ungauged (Target) location useful for design purposes: in particular, only geographical information and the knowledge of another gauged (Source) buoy are used. Several are the novelties introduced. (i) New hindcasting relations are derived. (ii) A full statistical model is set up for the Target area, whereas traditional hindcasting simply transfers time series from a gauged to an ungauged site: this gives the possibility to appropriately deal with design and hazard assessment at the Target location. (iii) The multivariate behavior of non-independent random variables is properly modelled by using the Theory of Copulas. As an illustration, a number of case studies is investigated, involving four pairs of buoys which, given their positions and exposures, are representative of a wide variety of sea states and conditions, as well as of different wave generation mechanisms.

## Introduction

Oceanographers and Maritime Engineers are primarily concerned with wave conditions at a specific location, in order to provide indications useful for the assessment of wave climate and the design of structures at sea at ungauged sites. The present work proposes a novel methodology to produce wave data at ungauged sites, based on historical data observed at a gauged site. In particular, the problem tackled here is: *what information about the sea state can be provided for design and hazard assessment at a Target location, if that site is ungauged?*

Hindcasting methods first appeared during World War II, when it became crucial to “translate” weather forecasts into expected wave conditions^1–3^. Briefly, wave hindcasting consists of using historical wind records and geographical information about the sea area of interest, in order to predict wave heights, periods and directions according to, e.g., wind duration and strength, and fetches: see, e.g., the SMB empirical method^4,5^. Later, more refined hindcasting guidelines were provided by^6^ exploiting the results of the JONSWAP experiment^7^.

In general, wave hindcasting techniques require minimal input information and computational effort, and provide ready and handy estimates. In turn, they may represent a valuable alternative/complementary approach with respect to, e.g., global wave models such as those developed at NOAA/ECMWF^8,9^—which, however, remain somewhat limited concerning the processes involved in the wave generation. Note that the outputs of hindcasting procedures can always be checked against global wave models.

After about 50 years since the JONSWAP trial, here the corresponding wave hindcasting relations take a new life. The approach proposed entails several novelties—see “Methods”. On the one hand, a full (statistical) model is developed for the ungauged Target site—instead of simply generating a limited data set of the Target variables of interest. On the other hand, the case of non-independent variables is properly dealt with, by using the Theory of Copulas. Furthermore, new hindcasting relations are derived.

In the present work, an original *isotropic Buoy-Ungauged Generator* procedure (hereinafter, *iBUG*) is outlined, in order to provide valuable hindcasting estimates of the sea state at an ungauged (Target) buoy, only exploiting some information given by another (Source) gauged buoy and the Effective Fetches (a geographical information always available at any site). The procedure is of global worldwide applicability, and works under minimal (and “natural”) assumptions, such as that the climatic event generating the sea storms has the same (comparable) features at both the Source and the Target sites. Note that also the predictions of global wave models implicitly thrust a “homogenous” sea state within each cell of the simulation grid used.

Here, a pragmatic approach is adopted. The idea is to check whether the iBUG procedure is able to provide valuable Target estimates of the design values of some relevant variables associated with given Return Periods (hereinafter, *RP*). The rationale is that such values are those used in practice for the design of structures at sea and the assessment of the corresponding hazards. In particular, in the present work, the Significant Wave Height *H* (in *m*) and the Sea Storm Duration *D* (in *h*) are used as the variables characterizing a sea storm—see “Materials”.

For this purpose, several buoys, subject to different sea state conditions and exposures, are considered on a pair-wise basis, and the following calculations are carried out.

1. Firstly, one buoy (say, A) is regarded as the Source of information (i.e., it is gauged), whereas another buoy (say, B, the Target one) is assumed to be ungauged (i.e., no data collected at the corresponding site are used to compute the iBUG estimates). Then, the iBUG procedure is utilized to set up a statistical model at B only using (i) the information available at A (i.e., the local observations of the variables *H* and *D*), and (ii) the ratios of the Effective Fetches at A and B. Finally, iBUG sea storm scenarios are generated at B via Monte Carlo techniques, and suitable *H* and *D* design values (associated with given RP’s) are compared with those actually observed at B.
2. The role of the buoys A and B is swapped: B is taken as the Source buoy, and A becomes the Target buoy. Then, the same calculations as above are carried out.

Most importantly, note that *only* the Source data, and the information about the Source and Target Fetches, are used to compute the iBUG Target estimates, since the Target buoy is assumed to be ungauged. This corresponds to a situation of “complete ignorance” of the sea state at the Target site, typical of many practical cases. The “double-blind” estimating approach used here (i.e., A vs. B, and B vs. A) gives the possibility to carry out an accurate roundtrip survey of the performance of the iBUG technique.

## Results and discussion

In this section, the outcomes of the analyses are illustrated and commented. Hereinafter, the following acronyms are used: MC for Monte Carlo, GoF for Goodness-of-Fit, and CI for Confidence Interval.

**Figure 1:**
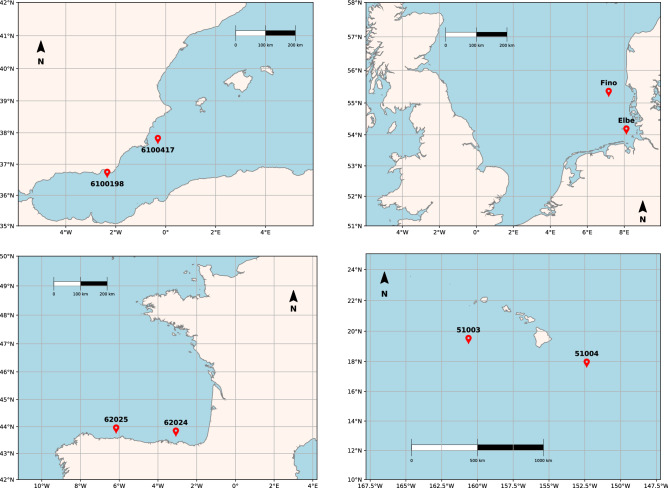
Maps. Locations of the pairs of buoys investigated. (*topleft*) The Gibraltar (Mediterranean Spain) case: buoys “6100417” and “6100198”. (top right) The North Sea case: buoys “Elbe” and “Fino”. (bottom left) The Bay of Biscay (Atlantic Spain) case: buoys “62024” and “62025”. (bottom right) The Hawaii (USA) case: buoys “51003” and “51004”.

In general, waves generated in deep water can be fetch-limited, duration-limited, or constitute a fully developed sea^10,11^. In order to thoroughly illustrate the applicability of the iBUG procedure, in the following four pairs of buoys A and B are considered: given their positions and exposures, these are representative of a wide variety of sea states and conditions, as well as of the wave generation mechanisms mentioned above. The features of the data sets are briefly introduced in “Materials”, and fully presented in the accompanying Supplementary Information (SI) document. The pairs investigated are the following: the corresponding maps are plotted in Fig. 1.

1. *Gibraltar (Mediterranean Spain).* The *Puertos del Estado* buoys “6100417” and “6100198”.
  URL: https://www.emodnet-physics.eu/Map
2. *North Sea.* The buoys located at *Elbe* (Federal Maritime and Hydrographic Agency-BSH, Germany) and *Fino* (R&D Centre, Kiel University of Applied Sciences, Germany).
  URL: https://www.emodnet-physics.eu/Map
3. *Bay of Biscay (Atlantic Spain).* The *Puertos del Estado* buoys “62024” and “62025”.
  URL: https://www.emodnet-physics.eu/Map
4. *Hawaii (USA).* The *NOAA* buoys “51003” and “51004”.
  URL: www.ndbc.noaa.gov

The figures presented in the sequel all have the same structure. Firstly, seven design Return Periods of practical interest are fixed: namely, 10, 20, 35, 50, 75, 100, and 200 years. Secondly, the two variables *H* and *D* are considered, and for each variable, 99% Confidence Intervals of the corresponding design values associated with the RP’s mentioned above are drawn. In particular: (i) *black* lines and markers are CI’s constructed using the Target data (the buoy B); (ii) *blue* lines and markers are CI’s constructed via Traditional Hindcasting techniques using the Source data (the buoy A); (iii) *red* lines and markers are CI’s constructed via the iBUG procedure using the Source data (the buoy A). In cases (i) and (ii), the CI’s are Bootstrap ones, while the iBUG CI’s are computed via Monte Carlo simulations—see “The iBUG procedure in practice”. Should two confidence intervals intersect, then it would not be (statistically) possible to reject the Null hypothesis that the corresponding point estimates of the design values are compatible (comparable). Below, the four case studies are presented and discussed.

**Figure 2:**
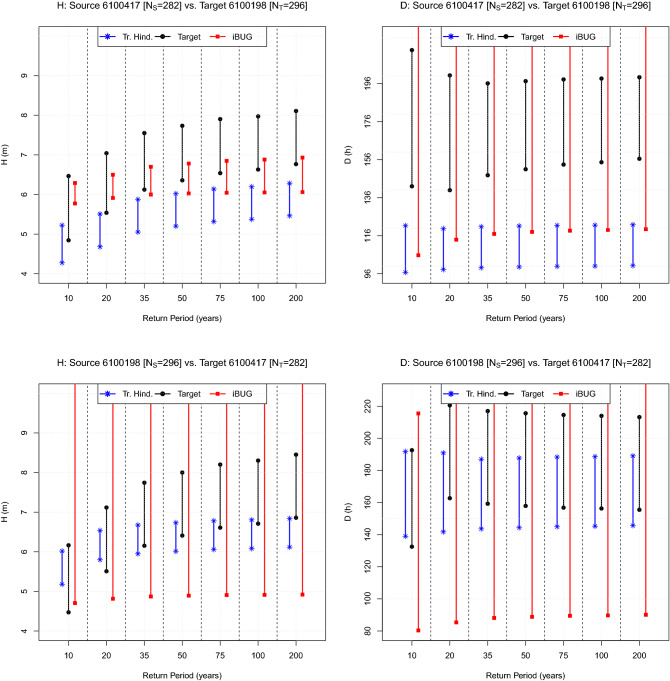
Comparison of Design values: the Gibraltar case. The plot shows the CI’s of the Target design values (black) associated with the RP’s indicated on the horizontal axis. The blue and red CI’s represent, respectively, the corresponding estimates computed via the Traditional Hindcasting and the iBUG techniques. The variables of interest (*H* and *D*) are indicated in the title, as well as the labels of the buoys considered and the Source ($N_{S}$) and Target ($N_{T}$) sample sizes.

### The Gibraltar case

The plots of interest are presented in Fig. 2. Considering the Source “6100417” buoy vs. the Target “6100198” buoy, the iBUG CI’s always intersect the Target ones, whereas the Traditional Hindcasting CI’s fail (at least) for RP’s larger than 20 years, both for *H* and *D*. Instead, swapping the two buoys (i.e., Source “6100198” vs. Target “6100417”), there are no failures, although the Traditional Hindcasting CI’s show kind of a limit approximation considering *H* and very large RP’s.

**Figure 3:**
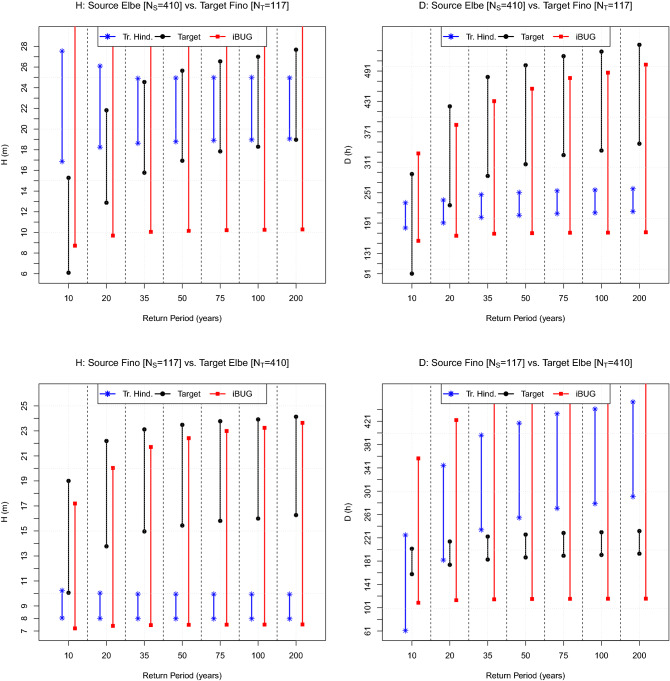
Comparison of Design values: the North Sea case. The plot shows the CI’s of the Target design values (black) associated with the RP’s indicated on the horizontal axis. The blue and red CI’s represent, respectively, the corresponding estimates computed via the Traditional Hindcasting and the iBUG techniques. The variables of interest (*H* and *D*) are indicated in the title, as well as the labels of the buoys considered and the Source ($N_{S}$) and Target ($N_{T}$) sample sizes.

### The North Sea case

The plots of interest are presented in Fig. 3. Considering the Source “Elbe” buoy vs. the Target “Fino” buoy, the iBUG CI’s always intersect the Target ones, whereas the Traditional Hindcasting CI’s fail (at least) for RP’s larger than 20 years, considering the duration *D*. Instead, swapping the two buoys (i.e., Source “Fino” vs. Target “Elbe”), there are no iBUG failures, whereas the Traditional Hindcasting CI’s fail, considering *H* and *D*, (at least) for RP’s larger than 20 years .

**Figure 4:**
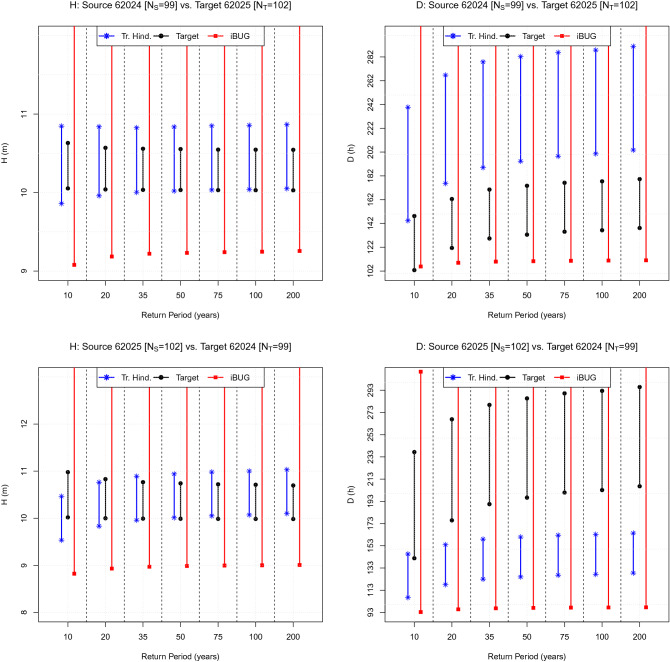
Comparison of Design values: the Bay of Biscay case. The plot shows the CI’s of the Target design values (black) associated with the RP’s indicated on the horizontal axis. The blue and red CI’s represent, respectively, the corresponding estimates computed via the Traditional Hindcasting and the iBUG techniques. The variables of interest (*H* and *D*) are indicated in the title, as well as the labels of the buoys considered and the Source ($N_{S}$) and Target ($N_{T}$) sample sizes.

### The Bay of Biscay case

The plots of interest are presented in Fig. 4. Considering the Source “62024” buoy vs. the Target “62025” buoy, the iBUG CI’s always intersect the Target ones, whereas the Traditional Hindcasting CI’s almost always fail, considering the duration *D*. Similarly, swapping the two buoys (i.e., Source “62025” vs. Target “62024”), there are no iBUG failures, whereas the Traditional Hindcasting fails considering the duration *D*.

**Figure 5:**
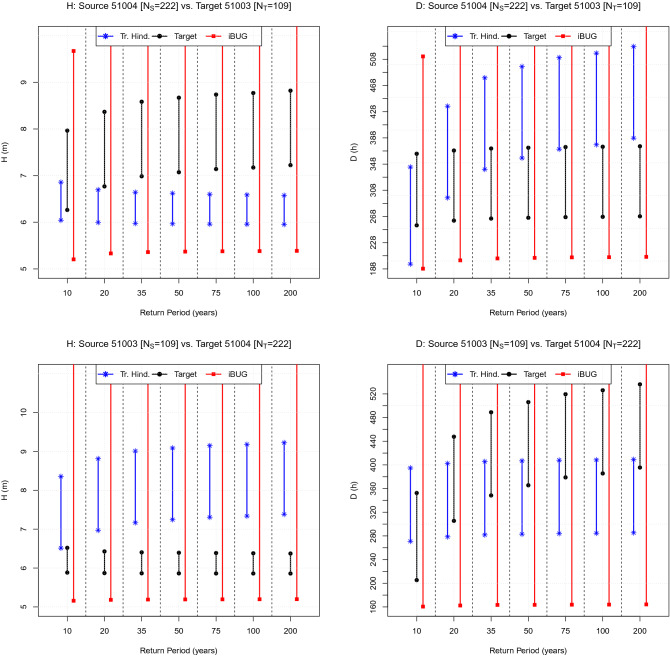
Comparison of design values: the Hawaii case. The plot shows the CI’s of the Target design values (black) associated with the RP’s indicated on the horizontal axis. The blue and red CI’s represent, respectively, the corresponding estimates computed via the Traditional Hindcasting and the iBUG techniques. The variables of interest (*H* and *D*) are indicated in the title, as well as the labels of the buoys considered and the Source ($N_{S}$) and Target ($N_{T}$) sample sizes.

### The Hawaii case

The plots of interest are presented in Fig. 5. Considering the Source “51004” buoy vs. the Target “51003” buoy, the iBUG CI’s always intersect the Target ones, whereas the Traditional Hindcasting CI’s almost always fail considering *H*. Instead, swapping the two buoys (i.e., Source “51003” vs. Target “51004”), there are no iBUG failures, whereas the Traditional Hindcasting CI’s almost always fail considering *H*.

### Interpretation of the results

As already mentioned above, the case studies considered in this work are representative of a wide variety of sea states and conditions, as well as of wave generation mechanisms.

The Traditional Hindcasting approach is nothing more than a *deterministic* technique: given a set of *N* data at a gauged Source buoy *S*, only a set of *N* estimates can be generated at a Target ungauged site *T*. Moreover, the Traditional Hindcasting may only yield design estimates based on a single sample (i.e., the data available at the Source buoy): in practice, only a single Target sea state scenario can be generated.

On the contrary, the iBUG procedure transfers a full statistical model at the target site *T*, i.e. it constructs a suitable Probability Space for the ungauged buoy, which also accounts for the dependence structure (i.e., the multivariate Copula) ruling the joint dynamics of the variables at play: note that this would not be possible via Traditional Hindcasting, being a deterministic univariate technique without any link to Statistics.

Most importantly, the statistical model used by the iBUG procedure may generate a number of possible (independent) Target scenarios via MC techniques: this entails that the Probability Space associated with the Target sea storms can be more appropriately and accurately explored, especially considering rare extreme events associated with large design Return Periods (difficult to observe via a single sample).

It is also interesting to note that, in general, the Monte Carlo iBUG CI’s are larger than the corresponding Target and Traditional Hindcasting ones, constructed via Bootstrap techniques. The reason is that the latter are based on a single sample of limited size *N*, whereas the iBUG CI’s represent the information extracted from many (independent) samples of size *N*—here, 100,000 MC simulations. Clearly, this gives the possibility to better account for the uncertainty and randomness of the Target sea states.

Overall, the results shown in “The Gibraltar case”–“The Hawaii case” indicate that the iBUG procedure is able to construct sea storm scenarios always compatible (comparable) with the sea state observed at the Target site (it is successful in 100% of the cases), whereas (as expected) the Traditional Hindcasting often fails: actually, in 9 cases out of 16, more than 50% of the tests, and especially considering Return Periods larger than 20 years (i.e., the ones of interest in practical applications).

## Materials

The available sea storm Time Series provide information about the significant wave height and the wave direction. In order to prevent problems with possible serial dependencies and correlations, in the present study an event-based approach is used (adopting a maritime version of the *Run Method* outlined in^12^), by considering suitable Sea Storm occurrences to describe the sea state.

Here, a sea storm is characterized via the Significant Wave Height *H* (in *m*) and the Sea Storm Duration *D* (in *h*), as well as a Direction parameter $\theta$ (in *deg N*): thus, the relevant triples are of type $(H,D; \theta )$—however, exploiting the multivariate approach outlined in this paper, other sets of variables can be considered, e.g. by using the Wave Period *P*, such as $(H,P; \theta )$, $(D,P; \theta )$, or $(H,D,P; \theta )$.

**Figure 6:**
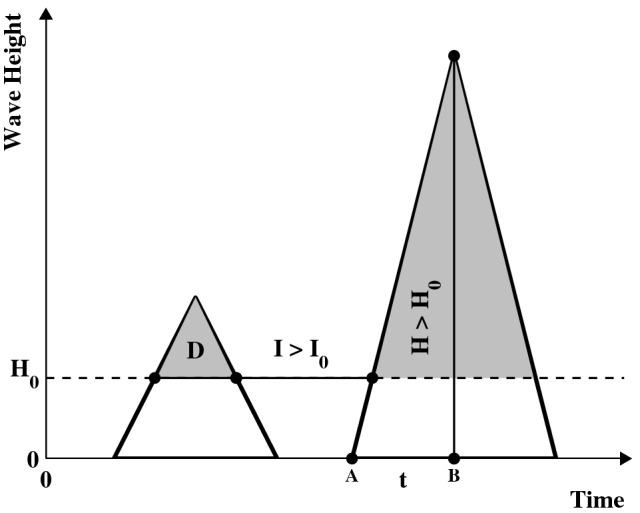
Triangular sea storm model. Graphical illustration of the Triangular Sea Storm model. Shown are: the wave height threshold $H_{0}$, the inter-storm period *I* larger than the threshold $I_{0}$, the sea storm duration *D*, a sea storm wave height *H* larger than $H_{0}$, and the approximate duration *t* of the climatic event generating the sea storm (the temporal segment ${\overline{AB}}$)—see text. The shaded regions indicate successive triangular sea storms.

Practically, a sea storm starts when *H* crosses upwards a given threshold $H_{0}$, and ends when *H* persists below the same level for at least $I_{0}$ hours: for a graphical illustration see Fig. 6, showing a standard Triangular Wave Model^13^ used to identify a sea storm sequence.

Here, the threshold $H_{0}$ corresponds to a suitable (large) percentile $p_{H}$ of the empirical distribution of wave heights, in order to focus the analyses on extreme events. The choice of the percentile represents a trade-off between (i) the purpose of investigating strong sea storms (and hence $p_{H}$ should be as large as possible) and (ii) the resulting size *N* of the extracted sample of sea storms (which should also be as large as possible for carrying out valuable statistical surveys): clearly, increasing $p_{H}$ reduces *N*, and vice-versa. In the present work, the following values of $p_{H}$ have been used: 95% for the Gibraltar case, 90% for the North Sea case, 95% for the Bay of Biscay case, and 75% for the Hawaii case.

Also, a minimum inter-storm temporal distance $I_{0}$ is used to temporally separate successive sea storms: this guarantees the physical independence of the events of interest. Actually, according to the tests provided by^14,15^ and implemented in the R package npcp, the distributions of the variables *H* and *D* extracted show no temporal Change-Points (distributional stationarity). Here, $I_{0}=48$ h for the Hawaii (accounting for the specific meteorology of the site), and $I_{0}=24$ h in all the other cases.

Once the thresholds $H_{0}$ and $I_{0}$ are chosen, the duration *D* of a sea storm is immediately computed, while the significant wave Height is taken as the maximum *H* during the whole sea storm, in order to investigate its extreme dynamics—and, correspondingly, $\theta$ is taken as the direction associated with the maximum Height recorded.

As anticipated in “Results and discussion”, in the present study four pairs of buoys are investigated. The accompanying SI shows the time series of *H* and *D*, the corresponding univariate fits, the fits of bivariate families of Copulas, and the Effective Fetches—see Sects. S.1–S.4. Here, eight standard univariate laws have been tested (namely, Weibull, Exponential, Gamma, Lognormal, Chi-square, F, GEV, GPD), as well as a dozen of copulas families and their survival variants (namely, Frank, Clayton, Gumbel, Joe, Tawn, Galambos, Husler–Reiss, *t*-Student). As a result all fits, both univariate and multivariate, are valuable, since the MC p-values of the GoF tests are large. It is worth noting that, in all cases, *H* and *D* are fairly well associated (concordant): in fact, the Kendall’s $\tau$ and Spearman $\rho$ measures of association^16^ are large, with negligible p-values—this corresponds to the failure of a test of independence for the pair (*H*, *D*).

## Methods

In this section, the mathematical framework adopted is thoroughly outlined. The present work is of *methodological* nature and of global worldwide applicability.

### Multivariate hindcasting

For the sake of illustration, here the (multivariate) analyses will involve the variables *H* and *D*. However, the same procedures can straightforwardly be used by considering other ones. The general idea (and novelty) is to make use of the observations available at a gauged Source buoy *S*, and then exploit the hindcasting relations provided by the JONSWAP experiment^7^, in order to construct a multivariate statistical model (accounting for the dependence between *H* and *D*) at an ungauged Target site *T*. Most importantly, such a model can be used to simulate suitable time series and scenarios of sea storms at *T* (see “Multivariate hindcasting”): clearly, the latters can be of any desired size, even much larger than the size of the sample observed at *S*—e.g. appropriate for specific design and hazard assessment purposes at *T* (see below).

The basic assumption of hindcasting procedures is that the climatic event generating the sea storms has the same (viz., “comparable”) features at both the Source and the Target site. In turn, the wave climate at the Source buoy *S* is supposed to be the same as—or comparable to—the (unobserved) one at the Target buoy *T*. In practice, such a condition/constraint can only be approximately satisfied: thus, the hindcasting outcomes must be taken with care. Here, the inspiring engineering principle is that a crude prediction at the ungauged site is always better than no information at all.

Below, we use Copulas as a basic tool for dealing with a multivariate framework. Indeed, a convenient way to model the joint behavior of a set $X_{1},\ldots ,X_{d}$ of random variables is to utilize Copulas. A copula is a multidimensional function describing, and mathematically formalizing, the dependence structure ruling the joint random dynamics of the $X_{i}$’s. Note that, in Literature, it has been increasingly recognized that the use of a multivariate framework is practically mandatory in Maritime Engineering, given the fact that often (if not always) the variables at play are non-independent—see^17–19^, as well as the typical case studies presented in “Materials”.

Indeed, recent advances in Mathematics show how copulas may represent an efficient tool to investigate the statistical behavior of dependent variables. For a theoretical introduction to copulas, see^20–22^; for a practical approach, see^16,23,24^. In particular, elementary guidelines for using copulas in maritime/hydrological applications are outlined in^18,19,25–28^. Freeware R routines are provided and illustrated in^29,30^.

According to Sklar’s Theorem representation^31^, the joint distribution

$$\begin{aligned} {\mathbf {F}}_{1,\ldots ,d}(x_{1},\ldots ,x_{d}) = {\mathbf {P}}_{}\left( {X_{1} \le x_{1},\ldots ,X_{d} \le x_{d}}\right) \end{aligned}$$

of the $X_{i}$’s, with marginal distributions $F_{i}$’s, can be written as, for all $(x_{1},\ldots ,x_{d}) \in {\mathbf {R}}^{d}$,

1 $$\begin{aligned} {\mathbf {F}}_{1,\ldots ,d}(x_{1},\ldots ,x_{d}) = {\mathbf {C}}_{1,\ldots ,d}\left( {F_{1}(x_{1}),\ldots ,F_{d}(x_{d})}\right) , \end{aligned}$$

where ${\mathbf {C}}_{1,\ldots ,d}$ is the copula associated with the random vector $(X_{1},\ldots ,X_{d})$. Practically, a multivariate model ${\mathbf {F}}_{1,\ldots ,d}$ can be readily constructed by (i) fitting suitable univariate laws $F_{i}$’s on the marginals $X_{i}$’s, and (ii) fitting an appropriate copula ${\mathbf {C}}_{1,\ldots ,d}$ on the observed $(X_{1},\ldots ,X_{d})$’s.

### Hindcasting based on JONSWAP formulas

Based on the ratio of Effective Fetches at a Source and a Target site, the JONSWAP hindcasting formulas^11^ make it possible to determine the wave climate at a site where no observations are available. Clearly, the outcomes of this methodology (hereinafter, *Transfer Method (TM)*), as well as the ones of traditional hindcasting methods, must be taken with care.

Let $\theta$ be a given direction: for instance, $\theta = 0^{\circ }$ N indicates the North. The TM consists of the following directional relations, where the subscripts “*S*” and “*T*” label, respectively, the Source and Target variables of interest:

2 $$\begin{aligned} {H_{T}}/{H_{S}}= & {} \varrho _{\theta }^{1/2}, \end{aligned}$$

3 $$\begin{aligned} {P_{T}}/{P_{S}}= & {} \varrho _{\theta }^{1/3}, \end{aligned}$$

4 $$\begin{aligned} {t_{T}}/{t_{S}}= & {} \varrho _{\theta }^{2/3}, \end{aligned}$$

where *t* is the duration of the climatic event generating a sea storm, and

5 $$\begin{aligned} \varrho _{\theta } = F_{T}(\theta )/F_{S}(\theta ) \end{aligned}$$

is the ratio of the Target $F_{T}(\theta )$ and Source $F_{S}(\theta )$ Fetches associated with the direction $\theta$.

Incidentally, as a novelty, the use of a Triangular Wave Model (see “Materials” and Fig. 6) may provide an estimate of *t*. In fact, it may be reasonable to assume that *t* equals half the duration of the triangular sea storm: viz., the storm “grows” until the wind feeds it (i.e., the temporal segment ${\overline{AB}}$ in Fig. 6), and then fades away. In turn, via the elementary proportion $t \div H = {D/2} \div {(H - H_{0})}$, an approximate value of *t* can be computed as

6 $$\begin{aligned} t = \frac{D\, H}{2\, (H - H_{0})}. \end{aligned}$$

As a consequence, exploiting Eqs. (4)–(6), a (new) rough estimate of the Sea Storm Duration ratio can be derived as

7 $$\begin{aligned} {D_{T}}/{D_{S}} = \varrho _{\theta }^{2/3}. \end{aligned}$$

Similar relations can be gleaned by using different Sea Storm Wave models.

### The iBUG procedure

As a novelty with respect to traditional hindcasting methods, here we do not simply transfer data bases from a Source to a Target site. Rather, by using Eqs. (2)–(7), we show how to convey a full statistical model from a (gauged) Source buoy *S* to an (ungauged) Target one *T*. In particular, here we focus the attention on the pair (*H*, *D*): however, there are no theoretical limitations to consider other sets of variables.

The advantages of transferring a full statistical model, instead of a single set of data, are obvious: in fact, once the (multivariate) statistics of the sea storms at the Target buoy *T* is set up, it is then possible to generate Target scenarios and samples of any suitable size (e.g., via MC simulations—see below), and thus provide useful information for design and hazard assessment at the ungauged site.

Before proceeding, the following important point needs to be stressed. In the present work, we adopt an *isotropic invariance principle*: viz., the Source marginals and copula do not depend upon the direction $\theta$. Instead, the Target distributions will be ruled by the directional Fetch ratios $\varrho _{\theta }$’s—see, below, Eqs. (8), (9), and (11). Actually, there are no theoretical reasons for requiring such a directional distributional invariance, rather only empirical ones. In fact, without such an assumption, specific univariate laws and copulas should be fitted for each $\theta$. Clearly, this makes little sense in practical applications (even introducing a coarse discretization of the directions $\theta$’s) since the directional sample sizes would almost surely be insufficient to provide a statistically significant number of observations, both for fitting the Source marginals and copula, and for carrying out univariate and multivariate Goodness-of-Fit tests. Practically, the iBUG procedure starts with an unique Source statistical model, and “drives” it to the Target buoy by exploiting the directional information provided by the Fetches’ ratios.

Now, denoting by $G_{S}^{H}$ and $G_{S}^{D}$, respectively, the univariate marginal distributions of the Source sea storm variables $H_{S}$ and $D_{S}$, it is immediate to calculate the corresponding laws $G_{T}^{H}$ and $G_{T}^{D}$ of the Target variables $H_{T}$ and $D_{T}$:

8 $$\begin{aligned} G_{T}^{H}(x; \theta )= & {} {\mathbf {P}}_{\theta }\left( {H_{T} \le x}\right) = {\mathbf {P}}_{}\left( {\varrho _{\theta }^{1/2}\, H_{S} \le x}\right) = G_{S}^{H}\left( x\, \varrho _{\theta }^{-1/2} \right) , \end{aligned}$$

9 $$\begin{aligned} G_{T}^{D}(y; \theta )= & {} {\mathbf {P}}_{\theta }\left( {D_{T} \le y}\right) = {\mathbf {P}}_{}\left( {\varrho _{\theta }^{2/3}\, D_{S} \le y}\right) = G_{S}^{D}\left( y\, \varrho _{\theta }^{-2/3} \right) , \end{aligned}$$

for all $x,y \in {\mathbf {R}}$. Evidently, given a direction $\theta$, the Target distributions are re-scaled versions of the Source ones via the Fetch ratio $\varrho _{\theta }$.

Then, by using Sklar’s Theorem, Eqs. (8)–(9), and the isotropic invariance, the bivariate probability distributions ${\mathbf {F}}_{S}$ and ${\mathbf {F}}_{T}$ of the pair (*H*, *D*) of, respectively, the sea storms at the Source and Target buoys, can be written as

10 $$\begin{aligned} {\mathbf {F}}_{S}(x,y)= & {} {\mathbf {C}}_{}\left( {G_{S}^{H}(x),G_{S}^{D}(y)}\right) , \end{aligned}$$

11 $$\begin{aligned} {\mathbf {F}}_{T}(x,y; \theta )= & {} {\mathbf {C}}_{}\left( {G_{S}^{H}\left( x\, \varrho _{\theta }^{-1/2} \right) , G_{S}^{D}\left( y\, \varrho _{\theta }^{-2/3} \right) }\right) , \end{aligned}$$

for all $x,y \in {\mathbf {R}}$, where ${\mathbf {C}}$ is the copula associated with the Source pairs $(H_{S},D_{S})$’s. Evidently, one of the advantages of transferring a full statistical model is that it may provide a complete set of information concerning the joint random behavior of the sea storms at the Target buoy.

### Estimates of design values

Traditionally, in Maritime Engineering (as well as in Terrestrial Hydrology) design values are computed via a Return Period approach. First, a design RP is chosen (e.g., 20, 50, or 100 years, depending on the structure of interest), and then the associated design value(s) of the variable(s) of interest are estimated using the available data. In the following, the general notion of RP introduced in^25,27^ is adopted. Given an event *E*, the associated RP $T_{E}$ is

12 $$\begin{aligned} T_{E} = {\mu }/{{\mathbf {P}}_{}\left( {E}\right) }, \end{aligned}$$

where $\mu$ is the mean inter-arrival time of the events *E*’s in the time series considered. Note that $\mu$ provides the time unit (e.g., years) in which $T_{E}$ should be expressed.

Considering events like $E=\{X>x^{*}\}$—the ones of typical interest in applications—where $x^{*}$ is a suitable threshold for the variable *X* (e.g., *X* could be the Significant Wave Height *H* or the Duration *D*), it is easy to compute design values $x^{*}$ once a design RP $T^{*}$ is assigned. In fact, Eq. (12) would become $T^{*} = {\mu }/{{\mathbf {P}}_{}\left( {X > x^{*}}\right) }$, and inverting it yields

13 $$\begin{aligned} x^{*} = F_{X}^{-1}( 1 - \mu /T^{*} ), \end{aligned}$$

where $F_{X}$ is the distribution function of *X*.

### The iBUG procedure in practice

Below it is shown how to simulate Target sea storm scenarios via the iBUG technique.

1. A set of design Return Periods *T*’s is fixed: here, *T* equals 10, 20, 35, 50, 75, 100, 200 years.
2. The original Source data are used to extract a set of $N_{S}$ (directional) sea storms $(H_{S},D_{S}; \theta )$’s.
  Note that, adopting a Traditional Hindcasting approach, and using Eqs. (2) and (7), then $N_{S}$ estimates of the pairs $(H_{T},D_{T}; \theta )$’s at the Target site would be given by
  14 $$\begin{aligned} \left\{ \begin{array}{l} {\tilde{H}}_{T,i} = \varrho _{\theta _{i}}^{1/2}\, H_{S,i}\\ {\tilde{D}}_{T,i} = \varrho _{\theta _{i}}^{2/3}\, D_{S,i} \end{array} \right. , \end{aligned}$$
  with $i \in 1,\ldots ,N_{S}$, and no further steps would be required.
3. Suitable univariate marginals $G_{S}^{H}$ and $G_{S}^{D}$, and a copula ${\mathbf {C}}$, are fitted over the Source data base, irrespectively of the different directions $\theta$’s associated with the sea storms, due to the isotropic invariance. As a statistical law for the directions $\theta$’s, the corresponding empirical distribution function ${\hat{F}}_{\theta }$ is used.
4. A set of $N_{S}$ pairs $(u_{i},v_{i})$’s is simulated from the Source copula ${\mathbf {C}}$. Similarly, a corresponding independent set of $N_{S}$ directions $\theta _{i}$’s is extracted from ${\hat{F}}_{\theta }$.
5. Via Sklar’s Theorem, and the Probability Integral Transform (i.e., by inverting $G_{T}^{H}$ and $G_{T}^{D}$), a sample (*x*, *y*)’s of $N_{S}$ iBUG Target pairs $(H_{T},D_{T};\theta )$’s is generated as
  15 $$\begin{aligned} \left\{ \begin{array}{l} x_{i} = \left( G_{T}^{H} \right) ^{-1} (u_{i}) = \varrho _{\theta _{i}}^{1/2}\, \left( G_{S}^{H} \right) ^{-1} (u_{i}) \\ y_{i} = \left( G_{T}^{D} \right) ^{-1} (v_{i}) = \varrho _{\theta _{i}}^{2/3}\, \left( G_{S}^{D} \right) ^{-1} (v_{i}) \end{array} \right. , \end{aligned}$$
  with $i=1,\ldots ,N_{S}$, where $\left( G_{\bullet }^{H} \right) ^{-1}$ and $\left( G_{\bullet }^{D} \right) ^{-1}$ denote, respectively, the inverses of the distribution functions $G_{\bullet }^{H}$ and $G_{\bullet }^{D}$. Thus, the pairs (*x*, *y*)’s represent a possible Target sea storm scenario according to the iBUG method.
6. Steps 4–5 are repeated *K* times, in order to provide a suitable number of independent Monte Carlo simulations: *K* may be chosen according to the desired accuracy of the iBUG estimates at the Target site for design and hazard assessment purposes (e.g., $10^{5}$ for large Return Periods, as in the present work).
7. Finally, using Eq. (13), and the *K* independent iBUG samples generated at Step 6, a set of *K* design values $x^{*}$’s and $y^{*}$’s is computed for each design RP *T* chosen at Step 1. In turn, appropriate Confidence Intervals can be constructed for the iBUG estimates. Similarly, via suitable Bootstrap techniques^32–34^, apt CI’s can also be set up for the corresponding Target and Traditional Hindcasting design values.

## Supplementary information

Supplementary Figures.

## Data Availability

The data used are available at the sites indicated in the paper, and specific requests may be sent to the Corresponding Author.
