# Supplementary material for: Multivariate sea storm hindcasting and design: the isotropic buoy-ungauged generator procedure
Source: Sci Rep. 2020 Nov 25;10:20517. doi: 10.1038/s41598-020-77329-y (PMC7689496; doi:10.1038/s41598-020-77329-y)
Supplement: Supplementary file 1 — Supplementary Figures. [file 41598_2020_77329_MOESM1_ESM.pdf]

Multivariate sea storm hindcasting and design: the isotropic Buoy-Ungauged Generator procedure

Gianfausto Salvadori[1,*], Giuseppe Roberto Tomasicchio[2], Felice D'Alessandro[3], Letizia Lusito[2], and Antonio Francone[4]

[1]Università del Salento, Dipartimento di Matematica e Fisica, Lecce, I-73100, Italy
[2]Università del Salento, Dipartimento di Ingegneria dell'Innovazione, Lecce, I-73100, Italy
[3]Università degli Studi di Milano, Dipartimento di Scienze e Politiche Ambientali, Milano, I-20133, Italy
[4]Università della Calabria, Dipartimento di Ingegneria Civile, Arcavacata di Rende, I-87036, Italy
[*]Corresponding Author: gianfausto.salvadori@unisalento.it

ABSTRACT

This document contains additional information concerning the data investigated in the main paper.

SUPPLEMENTARY INFORMATION

All the figures presented in the sequel share the same structure, and provide the same information for all the eight buoys considered. The legend is as follows. In the panels, N indicates the sample size.

1. (*topleft*) Time series of the observed H's.
2. (*topright*) Time series of the observed D's.
3. (*middleleft*) Fit of the observed H's: also indicated is the Monte Carlo p-value of a Kolmogorov-Smirnov Goodness-of-Fit test.
4. (*middleright*) Fit of the observed D's: also indicated is the Monte Carlo p-value of a Kolmogorov-Smirnov Goodness-of-Fit test.
5. (*bottomleft*) Copula fit of the observed pairs (H,D)'s: also indicated are the copula family, a Maximum Likelihood estimate of the parameter θ, and the Monte Carlo p-value of a Cramér-von Mises Goodness-of-Fit test.
6. (*bottomright*) Effective Fetches (in *km*).

   The next figure shows the locations of the eight buoys considered.

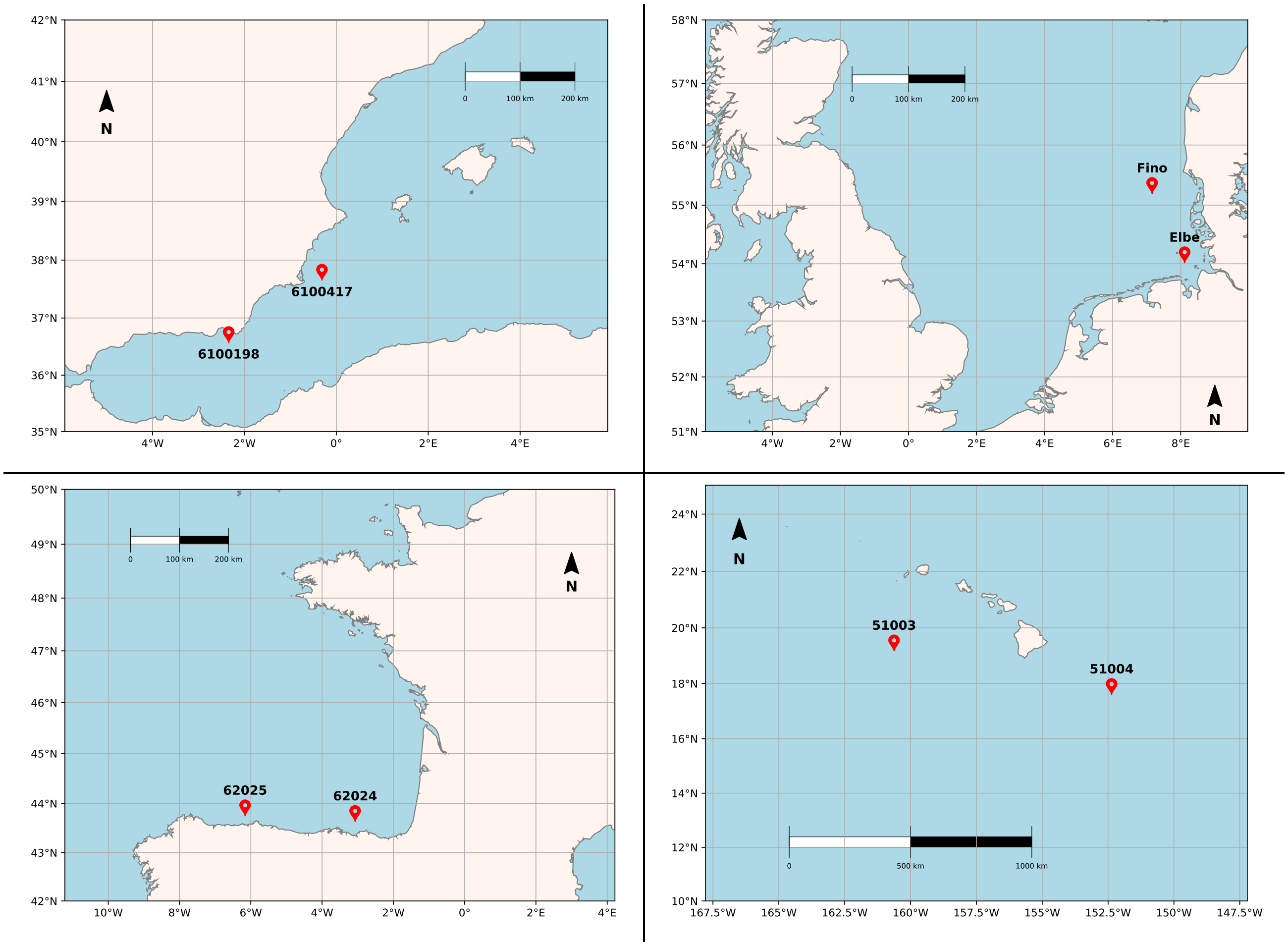

Figure S.0.1. Maps. Locations of the pairs of buoys investigated. (*topleft*) The Gibraltar (Mediterranean Spain) case: buoys "6100417" and "6100198". (*topright*) The North Sea case: buoys "Elbe" and "Fino". (*bottomleft*) The Bay of Biscay (Atlantic Spain) case: buoys "62024" and "62025". (*bottomright*) The Hawaii (USA) case: buoys "51003" and "51004".

S.1 The Gibraltar case (Mediterranean Spain)

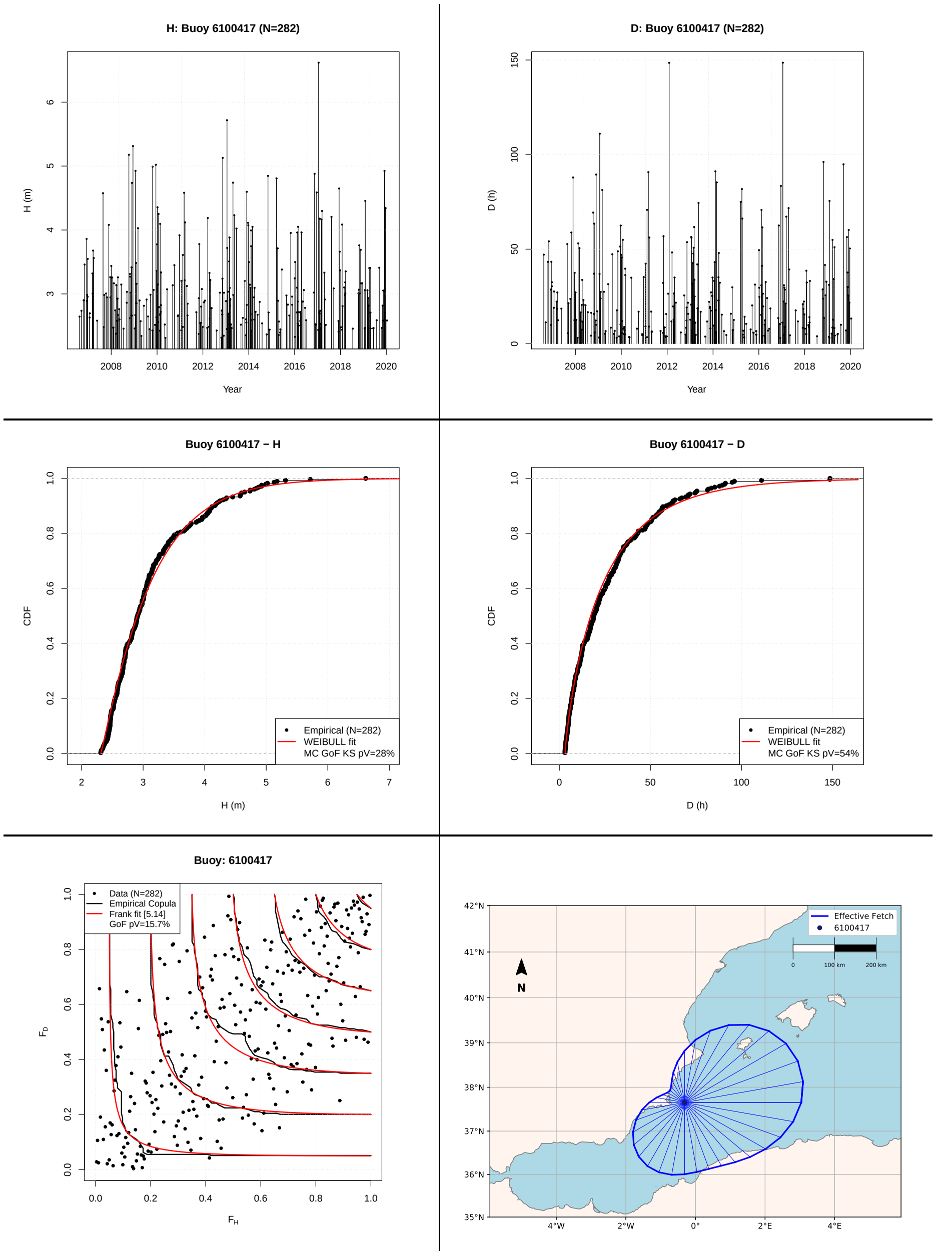

Figure S.1.1. The buoy “6100417”.

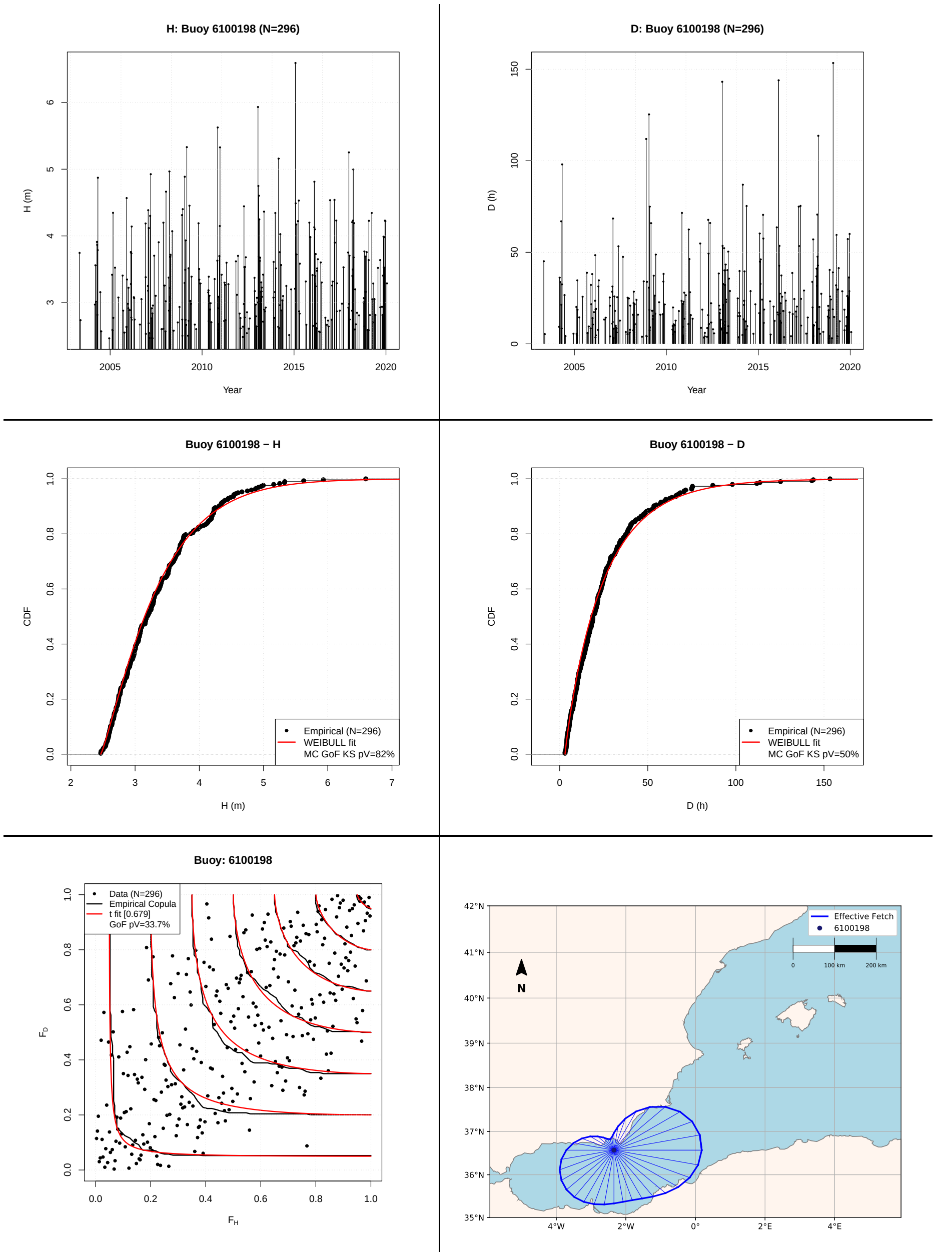

Figure S.1.2. The buoy “6100198”.

S.2 The North Sea case

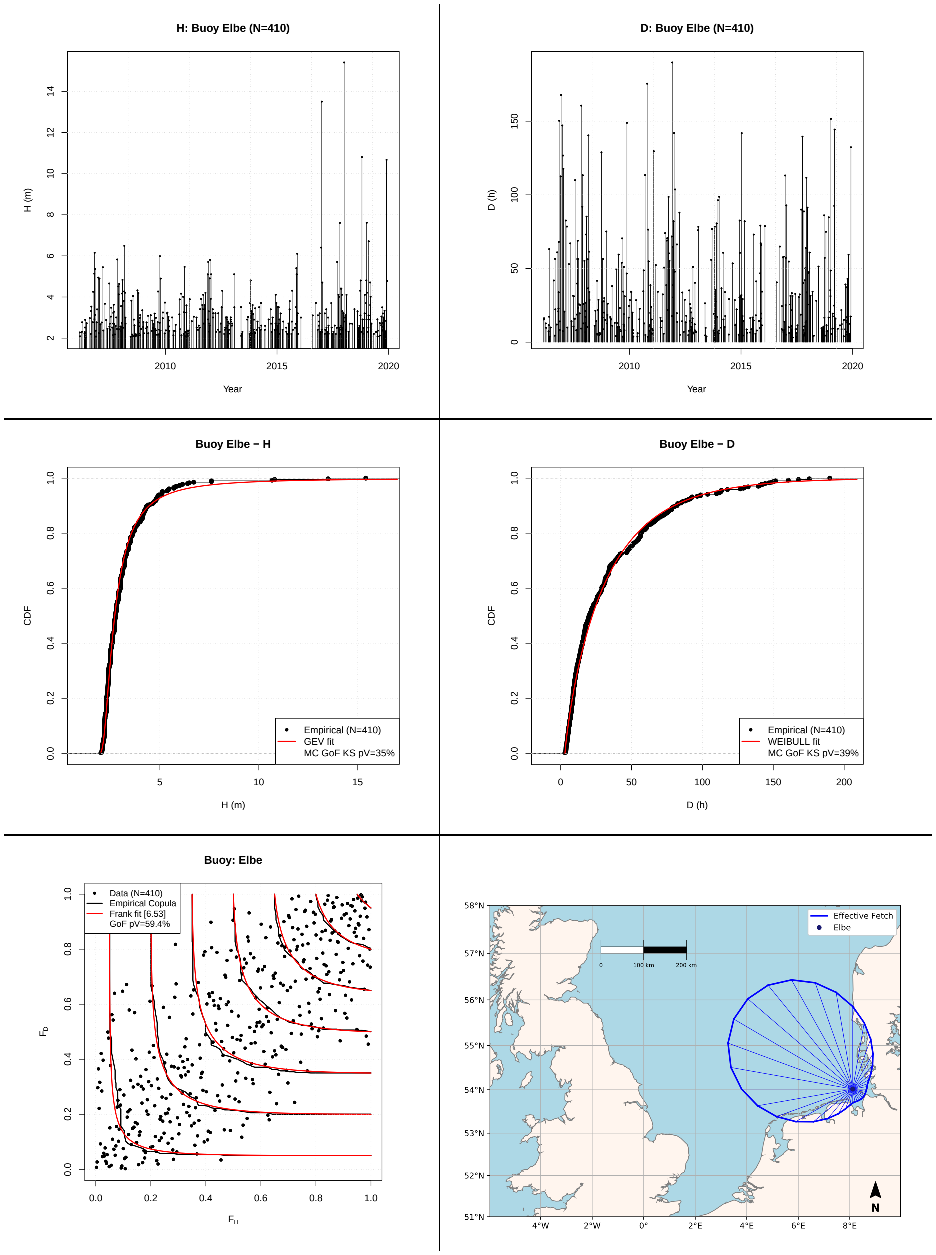

Figure S.2.1. The buoy “Elbe”.

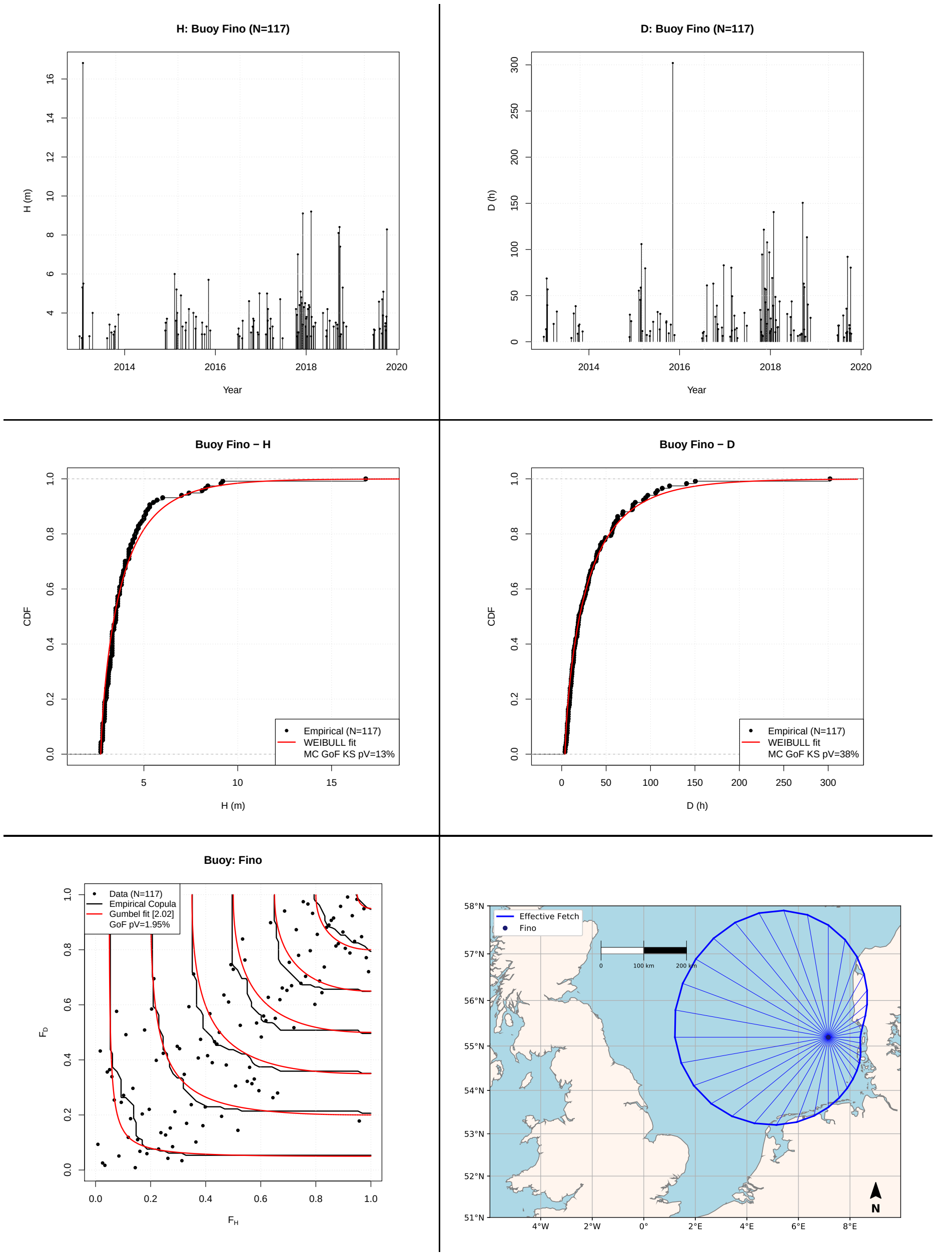

Figure S.2.2. The buoy “Fino”.

S.3 The Bay of Biscay case

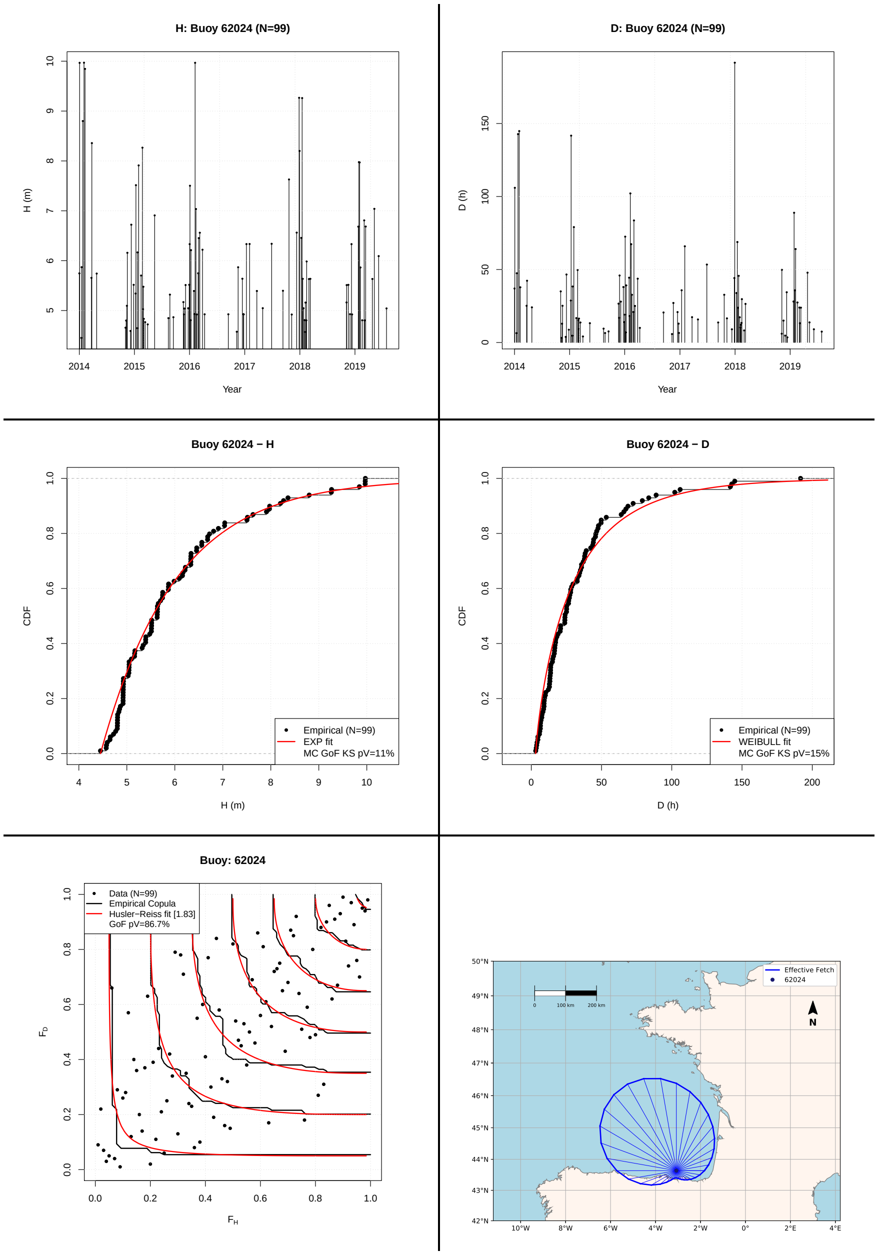

Figure S.3.1. The buoy “62024”.

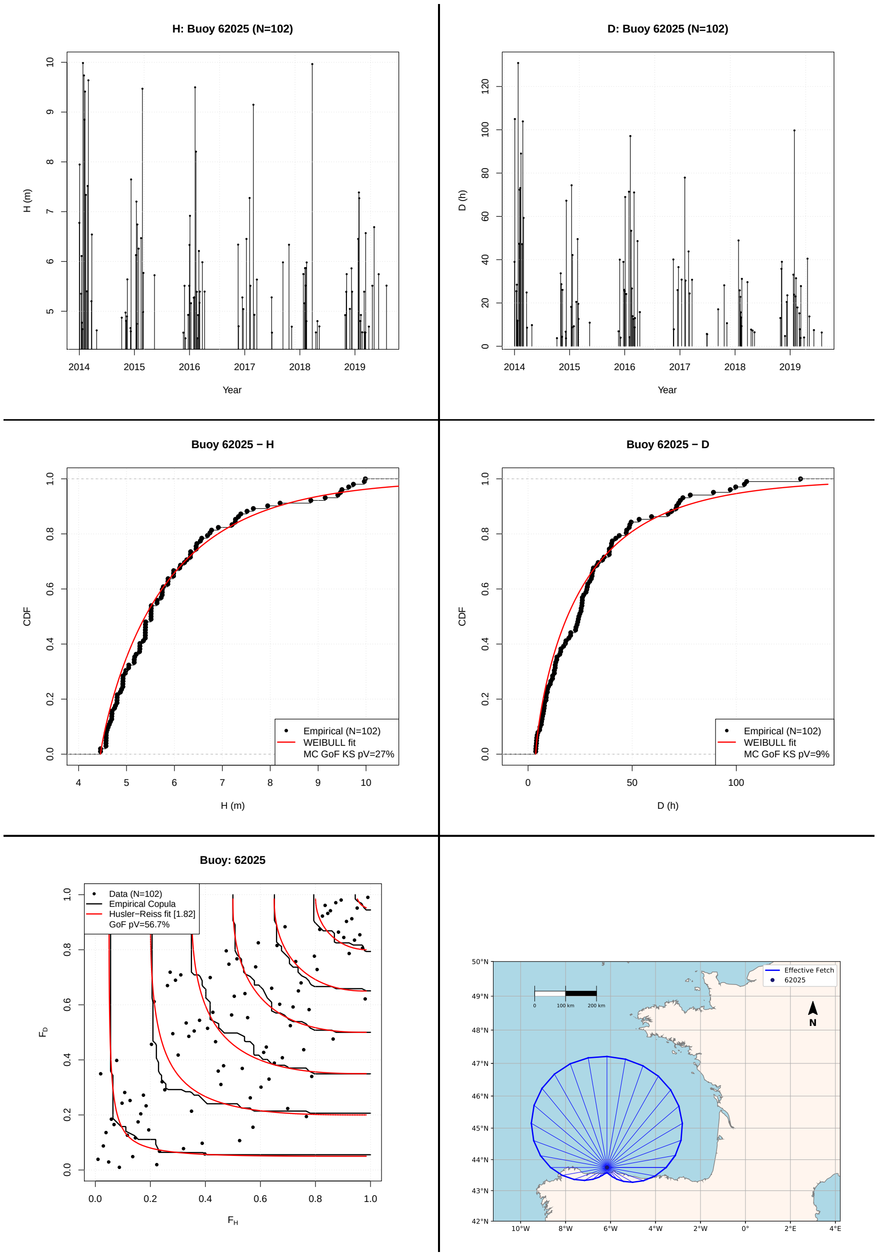

Figure S.3.2. The buoy “62025”.

S.4 The Hawaii case

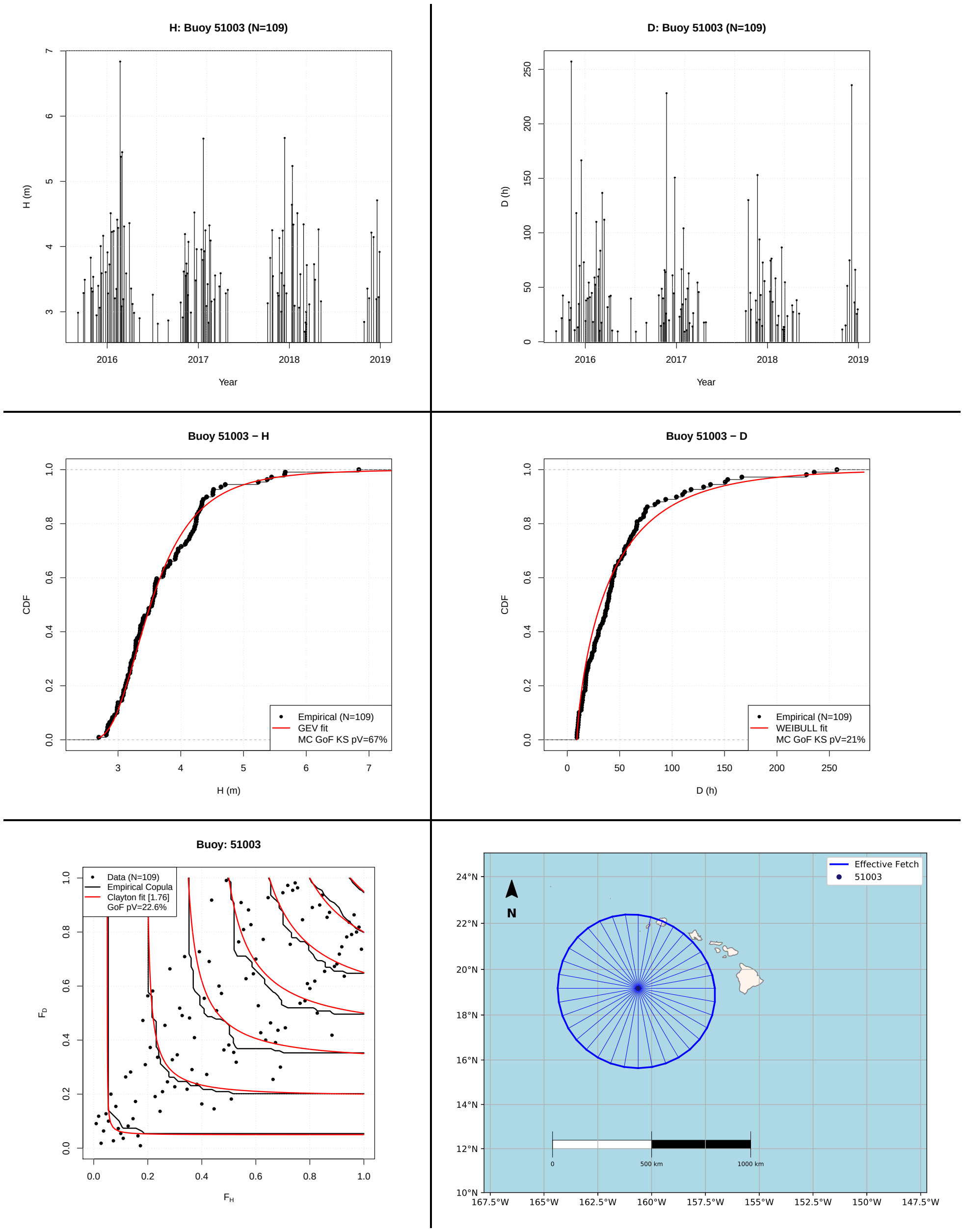

Figure S.4.1. The buoy “51003”.

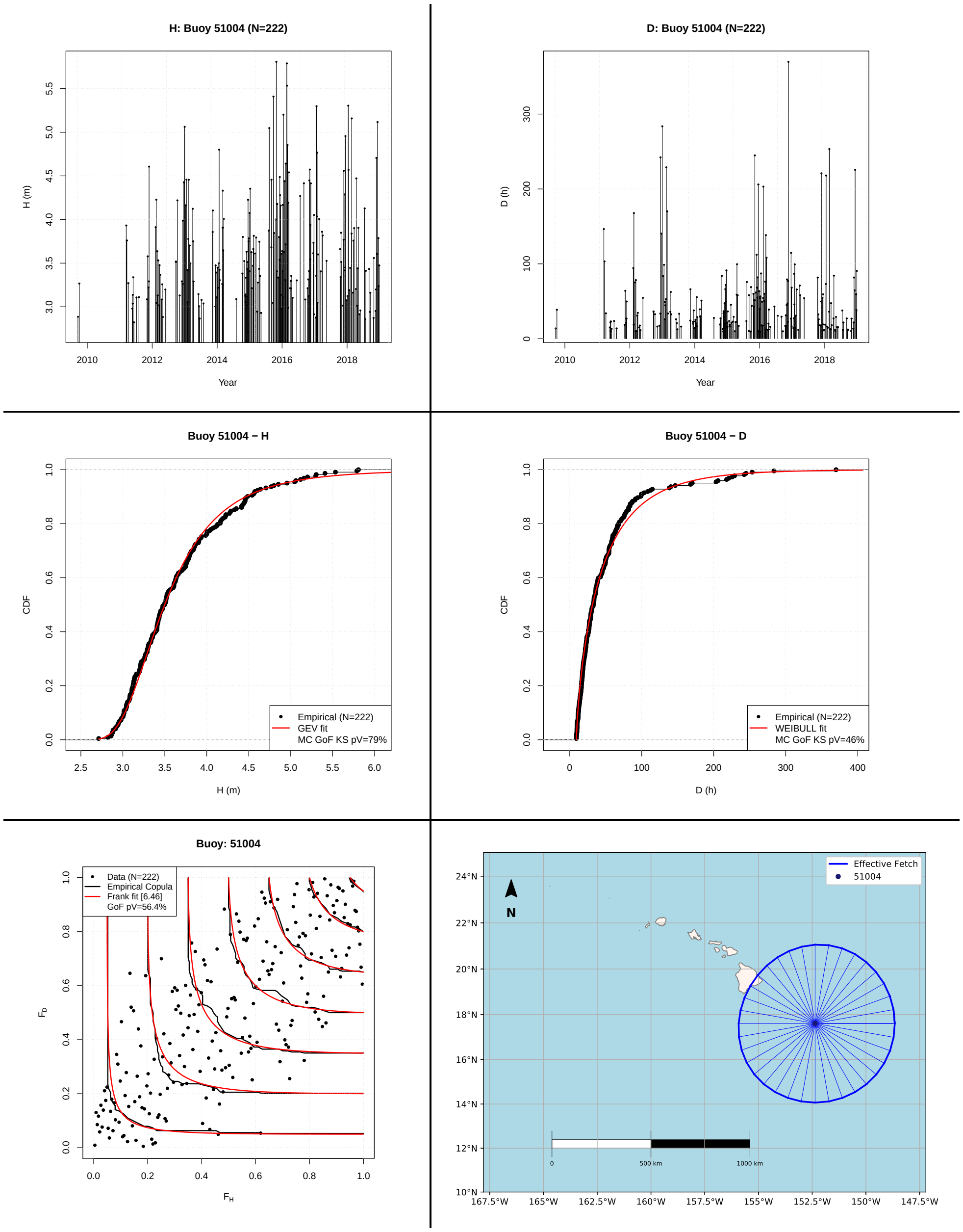

Figure S.4.2. The buoy “51004”.
